# Single-cell transcriptomic profiling of dorsal root ganglion: an overview

**DOI:** 10.3389/fnana.2023.1162049

**Published:** 2023-06-19

**Authors:** Keyu Xie, Xu Cheng, Tao Zhu, Donghang Zhang

**Affiliations:** ^1^Department of Anesthesiology, West China Hospital, Sichuan University, Chengdu, China; ^2^Department of Anesthesiology, Chengdu Second People’s Hospital, Chengdu, China

**Keywords:** DRG, scRNA-seq, snRNA-seq, neuronal types, review

## Abstract

The somatosensory neurons in the dorsal root ganglion (DRG) are responsible to detect peripheral physical and noxious stimuli, and then transmit these inputs into the central nervous system. DRG neurons are composed of various subpopulations, which are suggested to respond to different stimuli, such as mechanical, thermal, and cold perception. For a long time, DRG neurons were classified based on anatomical criteria. Recently, single-cell (scRNA-seq) and single-nucleus RNA-sequencing (snRNA-seq) has advanced our understanding of the composition and functional heterogeneity of both human and rodent DRG neurons at single-cell resolution. In this review, we summarized the current literature regarding single-cell transcriptomic profiling of DRG to provide an integral understanding in the molecular transcriptomes, cell types, and functional annotations of DRG neurons in humans and rodents.

## Highlights

- Providing a systematical summary of the DRG neurons classification at the single-cell resolution and predicting their putative biological functions across species from rodents to primates.
- Clarifying the conservation and divergence in the single-cell transcriptomics atlas across species, which will provide guidance for translating results obtained in animal models to the clinic.
- Pointing out several important genes with sex differences in neuronal gene expression in the human DRG, which may facilitate the understanding of the molecular mechanism for different prevalence of disorders between males and females.

## Introduction

Dorsal root ganglion (DRG) neurons play a pivotal role in perceiving and discriminating diverse types of sensation to specific stimuli, including thermoception, mechanoreception, proprioception, nociception, and pruritoception (Abraira and Ginty, 2013; Usoskin et al., 2015; Gong et al., 2019; Parpaite et al., 2021; Kanehisa et al., 2022). Different physical and noxious stimuli are detected by the specific type of DRG neurons, and the generated inputs are transmitted into the dorsal spinal cord (Todd, 2010; Li et al., 2016; Gatto et al., 2019). Based on the cell diameter, DRG neurons were classified into small and large populations with different functions (Ju et al., 1987; Basbaum et al., 2009; Li et al., 2011, 2016; Chen et al., 2020; Kanehisa et al., 2022). On the other hand, neuronal types are also usually classified according to the established markers by immunofluorescence or *in situ* hybridization methods (Bautista et al., 2007; Mishra and Hoon, 2013; Usoskin et al., 2015). This biased classification of types, the absence of markers for their identification, as well as the populations identified by different markers across species can hinder the functional identification, such as perception of touch, itch, pain, temperature and proprioception of specific neuronal type (Usoskin et al., 2015).

Single-cell RNA-sequencing (scRNA-seq) and single-nucleus RNA-sequencing (snRNA-seq) emerged in recent years, which enables unsupervised grouping of similar expression profiles to reveal cell populations of DRG and generates molecular architecture by capturing the whole cell or cell nucleus to perform RNA-sequencing at single-cell resolution (Usoskin et al., 2015; Hu et al., 2016; Li et al., 2016; Zhao et al., 2023). Recently, studies have identified the neuronal subtypes as well as their function aspects in mouse and primates DRG using scRNA-seq and/or snRNA-seq (Renthal et al., 2020; Kupari et al., 2021; Tavares-Ferreira et al., 2022; Jung et al., 2023). More importantly, the single-cell transcriptonic data have been compared across species (Kupari et al., 2021; Nguyen et al., 2021; Jung et al., 2023). Several studies used scRNA-seq and snRNA-seq to further explore the transcriptional changes in response to injury in the DRG (Hu et al., 2016; Renthal et al., 2020; Nguyen et al., 2021; Wang et al., 2021), which may identify new cell subtypes and discover gene regulatory networks associated with chronic pain, itch, and other somatosensory disorders associated with DRG neurons.

Herein, we reviewed the current literature about the single-cell transcriptomic profiling of DRG neurons across species, and summarized the component diversity as well as their functional heterogeneity. This review also clarified the conservation and divergence in the single-cell transcriptomics atlas across species, as well as revealed several important genes with sex differences in the DRG, which will provide some guidance for future studies about the molecular mechanism of somatosensory disorders and help translate results obtained in animal models to the clinic.

## Mouse neuronal types

**FIGURE 1:**
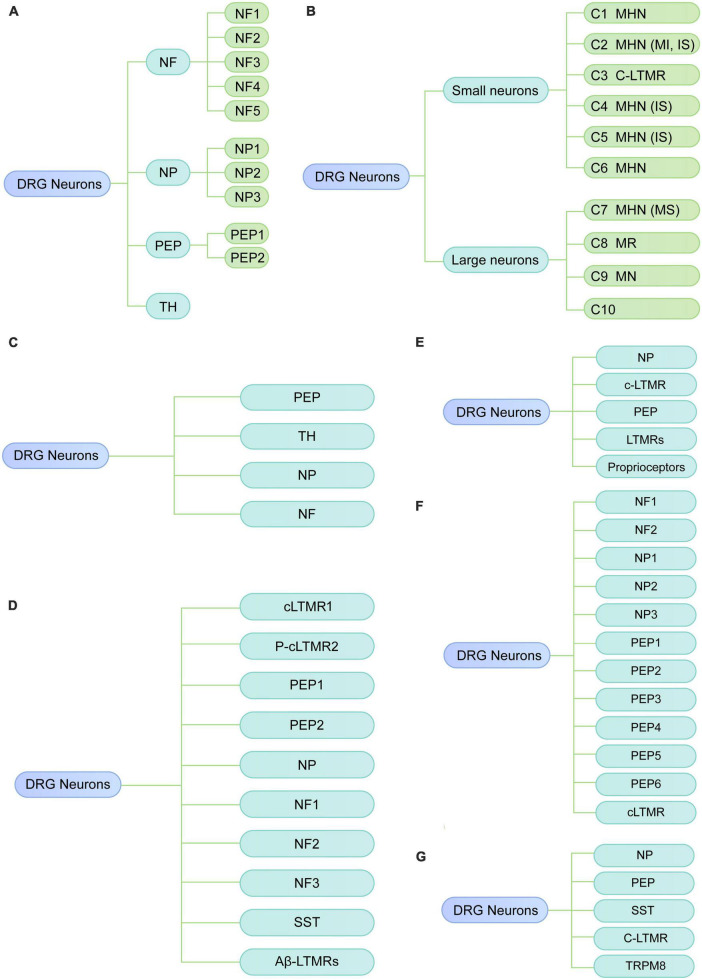
Diverse classes of DRG neurons revealed by scRNA-seq or snRNA-seq in rodents under physiological conditions. **(A)** Neuronal subtypes of mouse DRG from the study of Usoskin et al. (2015). **(B)** Neuronal subtypes of mouse DRG from the study of Li et al. (2016). **(C)** Neuronal subtypes of mouse DRG from the study of Wang et al. (2021). **(D)** Neuronal subtypes of mouse DRG from the study of Renthal et al. (2020). **(E)** Neuronal subtypes of mouse DRG from the study of Hu et al. (2016). **(F)** Neuronal subtypes of mouse DRG from the study of Zhou et al. (2022). **(G)** Neuronal subtypes of rat DRG by Zhang et al. (2022). NF, neurofilament; PEP, peptidergic; NP, non-peptidergic; TH, tyrosine hydroxylase; MHN, mechanoheat nociceptor; MI, mechanically insensitive; IS, itch-sensitive; MS, mechanically sensitive; MN, mechanical nociceptor; MR, mechanoreceptor; C-LTMR, C-fiber low-threshold MR; SST, somatostatin.

Usoskin et al. (2015) performed low-coverage scRNA-seq (3,574 ± 2,010 genes per neuron) on the mouse lumbar DRGs. They identified 11 distinct neuronal clusters, including three low-threshold mechanoreceptive neurons [neurofilament (NF)1, NF2, and NF3], two proprioceptive (NF4 and NF5), and six principal types of thermosensitive [peptidergic (PEP) 1], itch sensitive [non-peptidergic (NP)1, NP2 and NP3], type C low-threshold mechanosensitive [tyrosine hydroxylase (TH)] and nociceptive neurons (PEP2) (Figure 1A). In addition, they identified a neuronal type (NP3) that was suggested to be responsible to chronic states of inflammatory itch, whereas NP1 and NP2 were related to cholestatic itch and acute itch, respectively. Therefore, their results illustrate the cellular complexity and molecular basis underlying somatic sensation. However, this low-coverage sequencing technique only detected a limited number of genes in a single neuron, which may lead to a transcriptional variation among DRG neurons and influence the precise identification of neuronal types. Moreover, the related function of the identified clusters has not been validated.

Li et al. (2016) used high-coverage scRNA-seq (10,950 ± 1,218 genes per neuron) in combination with *in vivo* whole-cell patch clamp recording to classify somatosensory neurons of the mouse DRG into 10 types (C1-C10). Based on cell size, these 10 major clusters can be reclassified into small-diameter neurons (C1-C6) and large-diameter neurons (C7-C10) (Figure 1B). By integrating transcriptomic, morphological and functional characteristics, small-diameter neurons were functionally annotated as one type of low-threshold mechanoreceptor (LTMR) and five types of mechanoheat nociceptors (MHNs), while large neurons were categorized into four nociceptor types, including neurexophilin 1-expressing MHNs and mechanical nociceptors (MNs) expressing BAI1-associated protein 2-like 1 (Baiap2l1). C8 and C10 were predicted to be mechanoreceptors and/or proprioceptors in their study, which is consistent with previous studies that proprioceptive neurons are large neurons (Giacobassi et al., 2020). Compared with the results of previous study (Usoskin et al., 2015), Li et al. (2016) also identified the well-known neuron types, such as LTMR and itch-sensitive clusters; however, they identified several neuron types (C6, C7, and C8) which have not been previously reported. Additionally, the clusters found in Li et al. (2016) were neuron size-based and their functional properties have been further validated with *in vivo* whole-cell patch clamp technique. This study provides new insights into the biological function of diverse somatosensory neurons in mouse DRG via functional annotations of neuron subtypes; therefore, their findings may serve as an important resource for studying somatosensory mechanisms.

## Single-cell transcriptomic changes response to neuropathic pain in mouse DRG

To explore the transcriptomic changes in individual DRG neuron related to neuropathic pain, Wang et al. (2021) performed scRNA-seq on mouse DRG after peripheral nerve injury. Under physiological conditions, DRG cells were classified into nine major types with distinct molecular markers, including neurons, satellite glial cells, Schwann cells, vascular smooth muscle cells, vascular endothelial cells, immune cells, fibroblasts, and red blood cells, and vascular endothelial cells, capillary. To explore the heterogeneity of DRG neurons (Figure 1C), as well as the transcriptomic changes under neuropathic pain conditions, DRG neurons were further reclassified into 19 subtypes, including 6 clusters of peptidergic neurons (clusters 1–6), 5 clusters of non-peptidergic neurons (clusters 7–11), 5 clusters of myelinated neurons types (clusters 12–16) and 3 spared nerve injury-induced neuron clusters (SNIICs). These three SNIICs characterized by the expression of *Atf3*/*Gal*/*Gfra3* (SNIIC1), *Atf3*/*Mrgprd* (SNIIC2) and *Atf3*/*Gal*/*S100b* (SNIIC3), were originated from *Cldn9*^+^/*Gal*^+^, *Mrgprd*^+^, and *Trappc3l*^+^ DRG neurons, respectively. Therefore, alterations of gene expression patterns can switch neuron types after SNI. They also identified altered gene expression in specific subtypes were associated with pain. For example, the cardiotrophin-like cytokine factor 1, which activates astrocytes in the spinal dorsal horn, was upregulated in SNIIC1 neurons and implicated in the SNI-induced mechanical allodynia. Their findings provide new insights into understanding the dynamic change of neuronal types and their underlying molecular mechanisms during the development of neuropathic pain. As examples, the *Clcf1* and genes that expressed in emerging neuron types may be associated with the temporal development of neuropathic pain, which needs to be validated in future studies.

Renthal et al. (2020) also explored the transcriptional reprogramming of DRG neuron subtypes after nerve injury. They performed snRNA-seq on lumbar DRGs from adult naive mice and mice with three nerve injury models, including spinal nerve transection (SpNT), sciatic nerve crush (Crush), or sciatic nerve transection + ligation (ScNT). In the naive DRG, neurons were classified into ten subtypes, including *Tac1*^+^/*Gpx3*^+^ peptidergic nociceptors (PEP1), *Tac1*^+^/*Hpca*^+^ peptidergic nociceptors (PEP2), *Mrgprd*^+^ NPs, *Sst*^+^ pruriceptors (SST), Nefh^+^ A fibers including Aβ low-threshold mechanoreceptors (Aβ-LTMRs) and Parvalbumin (*Pvalb*)^+^ proprioceptors (NF1, NF2), *Cadps2*^+^ Aδ-LTMRs (NF3), *Fam19a4*^+^/*Th*^+^ C-fiber LTMRs (cLTMR1), and a putative cLTMR2 (p_cLTMR2) cluster that expresses *Fam19a4*, and low levels of *Th* (Figure 1D). Axonal injury-induced a common transcriptional program across different neuronal subtypes. Finally, their findings also revealed that *Atf3* is necessary for axotomy-induced transcriptional reprogramming, axonal regeneration, and sensory recovery after injury.

Hu et al. (2016) performed scRNA-seq analysis of non-peptidergic nociceptors (NP), peptidergic nociceptors (PEP), c-LTMR, LTMRs and proprioceptors in mouse DRG at 3 days after sciatic nerve transection (SNT) (Figure 1E). They found nerve injury-response genes including novel regeneration associated genes were associated with neuronal development, protein translation and cytoplasm transportation. In addition, the expression of genes related to cell death, such as *Pdcd2* in a subset of NP was markedly increased after nerve injury.

Zhang et al. (2022) conducted scRNA-seq to characterize subtype-specific changes of transcriptomes in mouse lumbar DRG neurons under chronic constriction injury (CCI). They confirmed 12 well-known neuronal clusters as described by previous studies (Renthal et al., 2020; Wang et al., 2021), including NP1 (*Nppb*^+^ non-peptidergic nociceptors), NP2 (*Mrgprd*^+^/*Cd55*^+^ non-peptidergic nociceptors), NP3 (*Mrgpra3*^+^/*Cd55*^+^ non-peptidergic nociceptors), PEP1-2 (*Tac1*^+^/*Sstr2*^–^ peptidergic nociceptors), PEP3-4 (*Tac1*^+^/*Sstr2*^+^ peptidergic nociceptors), PEP5 (*Trpm8*^+^ peptidergic nociceptors), PEP6 (*Trpv1*^+^ peptidergic nociceptors), NF1-2 (*Nefh*^+^/*Scn1b*^+^ Aβ low-threshold mechanoreceptors), and cLTMR (*Fam19a4*^+^/*Th*^+^ C-fibers low-threshold mechano-receptive neurons) (Figure 1F). Additionally, their findings identified four CCI-induced clusters, which expressed high levels of injury-related genes such as *Sprr1a* and *Atf3*. Of note, their sequencing data showed that *Atf3* expression was not restricted to injured neurons (*Sprr1a*^+^) but also was detected in sham-operated neurons (*Sprr1a*^–^), which is consistent with the previous findings in rodents (Shortland et al., 2006; Nguyen et al., 2019). However, *Sprr1a* was selectively expressed in injured neurons of CCI mice, suggesting that *Sprr1a* may serve as a more specific marker of injured neurons than *Atf3*. Moreover, it should be remembered that *Sprr1a* is a species specific response since it is not up-regulated in rat DRGs after nerve injury (Starkey et al., 2009). After CCI, four clusters were identified, i.e., NP1, PEP5, NF1, and NF2 that showed different transcriptional patterns from other clusters. They further explored the sex differences in transcriptional changes after CCI. The overall transcriptional patterns were similar between male and female mice under physiologic conditions, as the subcluster distributions were similar and the cluster comparison showed good correlation between female and male mice. Nevertheless, many sex-specific differentially expressed genes (DEGs, 79 female-specific and 86 male-specific) were identified after CCI. Pearson correlation analysis showed a good correlation of DEGs between sex in most neuronal clusters, but a poor correlation in the cLTMR cluster, suggesting substantial divergence existed in the cLTMR cluster between sex. They further found *CALCA* (a nociceptor-specific gene) was differentially expressed in cLTMRs between sex, suggesting *CALCA* may be a molecular target to explain the different prevalence of pain between females and males. These findings also suggested that cLTMRs may represent important subpopulations in the sexual dimorphism of neuropathic pain. Lastly, they validated the expression, distribution as well as the function of *Fgf3* using *in situ* hybridization and *in vitro* calcium imaging study, highlighting that increased *Fgf3* in injured neurons contributes to the development of neuropathic pain. Their study provides an important resource of cell-subtype-specific and sex-specific genes related to neuropathic pain, which may contribute to developing more specific therapies for neuropathic pain.

## Rat neuronal types

Zhou et al. (2022) examined gene expression profiles of rat DRG by scRNA-seq. They identified eight major cell types, including neurons, satellite glial cells (SGC), proliferating SGC, Schwann cells, fibroblasts, vascular smooth muscle cells, vascular endothelial cells, and microglia. Neurons were reclassified into 11 subclusters, which were annotated as non-peptidergic nociceptors (NP), peptidergic nociceptors (PEP), somatostatin^+^ neurons (SST), C-fiber low-threshold mechanoreceptors (CLTMR), and *Trpm8*^+^ neurons (TRPM8) (Figure 1G). Furthermore, they found a novel neuron type (*Fxyd7*^+^/*Atp1b1*^+^) associated with mechanical allodynia (MA) in diabetic rats, which were suggested to originate from the peptidergic neuron cluster. Their study reveals the transcriptomic changes in DRG neurons at single-cell resolution in rats with diabetic peripheral neuropathy. For example, several genes involved in “sensory perception of pain” were downregulated, including *Trpv1*, *Asic1*, *Adcyap1*, *Trpa1*, *Npy1r*, *Oprk1*, and *Tac1*. However, they did not perform experiments to validate their findings.

## Primate neuronal types

**FIGURE 2:**
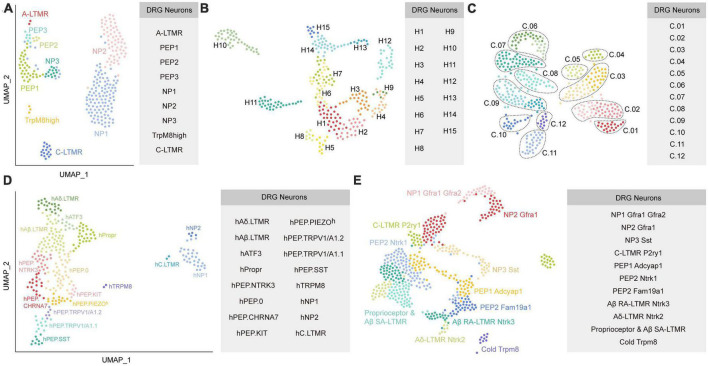
Diverse classes of DRG neurons revealed by scRNA-seq or snRNA-seq in primates. **(A)** Neuronal subtypes of macaque DRG from the study of Kupari et al. (2021). **(B)** Universal manifold (UMAP) showing the diverse transcriptomic classes of human somatosensory neurons from the study of Nguyen et al. (2021). **(C)** UMAP plot showing the human DRG neuronal clusters from the study of Tavares-Ferreira et al. (2022). C.01: proprioceptors; C.02: A SA LTMRs; C.03: A RA LTMRs; C.04: A LTMRs; C.05: putative A nociceptors; C.06: cold nociceptors; C.07: A HTMRs; C.08: PENK+ nociceptors; C.09: TRPA1+ nociceptors; C.10: putative silent nociceptors; C.11: pruritogen receptor enriched; C.12: putative C-LTMRs. **(D)** UMAP plot showing the human DRG neuronal clusters from the study of Yu et al. (2023). **(E)** UMAP plot showing Seurat-integrated DRG neuronal clusters across mouse, guinea pig, cynomolgus monkey, and human from the study of Jung et al. (2023). Each colored dot represents a cell (The same colors across a-e do not represent the same clusters). PEP, peptidergic; NP, non-peptidergic; A-LTMR, A-fiber low-threshold mechanoreceptors; C-LTMR, C-fiber low-threshold mechanoreceptors; SST, somatostatin.

Kupari et al. (2021) classified DRG neurons in non-human primate using scRNA-seq and identified their contributions to chronic pain by mapping human pain heritability to distinct neuronal types. DRG neurons from adult Rhesus macaques were divided into nine subclusters based on their distinct transcriptomics. Then, they defined these nine neuron types according to their mouse counterparts by examining the profiles of canonical markers of mouse DRG neuron. As a result, these nine subtypes were assigned as NP1, NP2, NP3, PEP1, PEP2, PEP3, C-LTMRs, TrpM8*^high^*, and A-LTMRs (Figure 2A). They further compared their transcriptional data with previously published mouse data (Zeisel et al., 2018; Sharma et al., 2020) and found overall similarity, although individual genes showed cell-type-specific differences. For example, *TDRD1* and *EDN3* specifically expressed in macaque NP3, whereas *NPPB* and *NTS* were specific in mouse NP3. For C-LTMRs, *TH* showed specifically high expression in mice, but *CCKBR* selectively expressed in macaques. These species differences may influence the translation of therapeutic targets from rodents models to primates. Their findings also suggested that NP1 and NP2 showed differential gene expression patterns between mice and macaques. Additionally, they mapped human genomic loci related to chronic pain onto primate neuronal subtypes to identify the potential interactions. The results revealed that PEP1 neurons had a higher association with headaches, facial, neck and shoulder, stomach, and hip chronic pains, while NP2 neurons were robustly related to the heritability of chronic back pain and hip pain. It will be meaningful to validate whether targeting PEP1 or NP2 improve these chronic pain conditions in future studies.

To explore the transcriptional profiling in human DRG, Nguyen et al. (2021) performed snRNA-seq on human lumbar 4-5 DRGs from one male and four female donors. Somatosensory neurons were classified into 15 clusters (Figure 2B). They further examined similarities and differences between neuronal clusters in humans and mice (Renthal et al., 2020). Although multiple homologous neuronal types exhibit similar transcriptomic features between species, expression of genes related to sensory function often was different. For example, *Tmem100*, which contributed to persistent pain in mice, was almost undetectable in the sequencing data of human DRG. *TRPM8*^+^ neurons in H8, a putative cool responsive cluster, also expressed high expression of *NTRK2* but not *TAC1*; however, rodents showed the opposite results. Furthermore, the sphingosine-1-phosphate receptor *S1PR3* that serves as a potential target for treating pain and itch in mice (Hill et al., 2018), was not markedly expressed in human DRG. In addition, the counterparts for several human clusters were not identified in the mouse. For instance, the gene expression pattern of H12 suggested it was different from any potential counterpart in mice. Interestingly, two clusters (H10 and H11) have transcriptomic features that resemble with the mouse non-peptidergic nociceptors NP1-3, which may mediate itch.

Tavares-Ferreira et al. (2022) performed spatial transcriptomics to characterize the molecular transcriptomes of DRG neurons from eight donors. Compared to scRNA-seq or snRNA-seq, spatial transcriptomics can provide spatial distribution information for the identified clusters. Furthermore, spatial transcriptomics will accurately sample cytoplasmic RNA and reveal the full neuronal transcriptome, which is important when detecting genes that have low expression. One important limitation for spatial transcriptomics is that its resolution is lower than that of scRNA-seq or snRNA-seq. Using spatial transcriptomics, Tavares-Ferreira et al. (2022) identified 12 clusters of human sensory neurons, including 5 C nociceptors, 1 C low-threshold mechanoreceptors (LTMRs), 1 Aβ nociceptor, 2 Aδ, 2 Aβ, and 1 proprioceptor subtypes (Figure 2C). They also provided a map of expression profiles for ion channels, G protein-coupled receptors (GPCRs), and other pharmacological targets in the human and mouse DRG (Zeisel et al., 2018). The comparison results reveled that multiple genes had similar expression patterns between humans and mice, such as the family of voltage-gated sodium channels. Intriguingly, the sodium voltage-gated channel beta subunit 4 (*SCN4B*) gene, which is important for neuronal excitability, was found mostly in mouse A-fiber neurons but was present in almost all sensory neuron types of humans. For GPCRs, many genes, such as *PTGER3* and *LPAR3*, showed divergent patterns of expression between humans and mice, indicating important heterogeneity in this family across species. Their findings also revealed the different expression patterns in interleukins (ILs) and their receptors between humans and mice. *IL31RA* was restricted to mouse itch nociceptors, but showed broad expression in human DRG neurons. IL-4 receptor (*IL4R*), IL-10 receptor subunit alpha (*IL10RA*), and IL-13 receptor subunit alpha 1 (*IL13RA1*) were more widely expressed across human DRG neuron subtypes than in mice. Strikingly, Il4r was not detected in mouse DRG neurons.

Moreover, they also compared the human findings to that from non-human primates and revealed a conserved expression pattern of genes among many subclusters but divergence in specific nociceptor across these two species. As examples, *PVALB* (Parvalbumin) expression was enriched in human proprioceptors and A SA LTMRs but not in their corresponding populations in macaques. Instead, *PVALB* was highly expressed in macaque PEP2 neurons (Kupari et al., 2021). Notably, the gene expression patterns of macaque C-LTMRs are divergent from all identified clusters in humans. This species difference needs to be considered in clinical translation. Finally, they identified sex differences regarding gene expression in specific subpopulations although same DRG neuronal subtypes existed in males and females. In their study, the pruritogen receptors clusters exhibited the highest number of differentially expressed genes (DEGs), suggesting sex-dependent molecular mechanisms. Of these DEGs, calcitonin-related polypeptide alpha (*CALCA*), which encodes the CGRP protein, is markedly increased in female pruritogen receptors clusters.

A recent preprint in bioRxiv (Yu et al., 2023) reported a novel method of isolating individual neuron soma from human DRGs for RNA-seq. As a result, 16 neuronal types were identified, including hTRPM8, hC.LTMR, hNP1, hNP2, hPEP.SST, hPEP.TRPV1/A1.1, hPEP.TRPV1/A1.2, hPEP.PIEZO, hPEP.KIT, hPEP.CHRNA7, hPEP.NTRK3, hPEP.0, hAδ.LTMR, hAβ.LTMR, hPropr, and hATF3 (Figure 2D). They further performed cross-species comparisons between their human transcriptome dada with previously published mouse (Sharma et al., 2020) and macaque datasets (Kupari et al., 2021), and highlighted several interesting species differences. For example, PEP1.1/CGRP-α and PEP1.2/CGRP-β clusters in mice were not detected in human. Furthermore, the counterparts of human hPEP.PIEZO*^h^*, hPEP.NTRK3, and hPEP.0 were absent in mouse and macaque datasets. Additionally, some genes showed divergent expression patterns in the homologous clusters across species. For instance, mechanosensory transduction mediator homolog (*STUM*) and calsequestrin 2 were specifically expressed in h.TRPM8 and hC.LTMR, respectively, whereas they were not specific in the counterparts of macaque and mouse. Finally, they predicted functional properties for some human clusters according to their transcriptomes and validated them by single-cell *in vivo* electrophysiological recordings. For example, hPEP.SST afferents were suggested implicated in itch sensation and inflammatory conditions, but were not associated with mechanosensitivity. The hTRPM8 population was supposed to function in cold, heat, and chemical sensations. In general, neuron types that associated with touch-, cold-, and itch-sensing were relatively conserved across species, while the pain-sensing related clusters exhibited notable divergence. Their datasets provided a valuable map for human somatosensation, which will serve as an important resource for clinical translation from animal results.

Although previous studies have compared their human and/or non-primate transcriptome atlas with the mouse data from other studies, differences in sequencing platforms, laboratory protocols, or sample processing methods between studies make it challenging to precisely integrate transcriptomic data from different species and to interpret the comparison findings. To overcome this shortcoming, a recent study (Jung et al., 2023) generated a transcriptome atlas of mouse, guinea pig, cynomolgus monkey, and human DRGs to directly determine the similarities and differences across species. snRNA-seq yielded 17 distinct neuronal subtypes in mouse DRGs based on canonical markers, including proprio & Aβ SA, Aβ RA, Aδ, NP1.1, NP1.2, NP1.3, NP2.1, NP2.2, NP3, PEP1.1, PEP1.2, PEP2.1, PEP2.2, PEP2.3, C-LTMR1, C-LTMR2, and cold clusters. To make a direct comparison between homologous clusters across species, they integrated DRG neurons from mouse, guinea pig, cynomolgus monkey, and human using Seurat, which is commonly conducted to aggregate single-cell RNA-seq datasets using anchors identified from canonical correlation analysis (Stuart et al., 2019). These integrated DRG neurons were classified into 11 subtypes, namely proprioceptor & Aβ SA-LTMR, Aβ SA-LTMR *Ntrk3*, Aδ-LTMR *Ntrk2*, C-LTMR *P2ry1*, NP1 *Gfra1 Gfra2*, NP2 *Gfra1*, NP3 *Sst*, PEP1 *Adcyap1*, PEP2 *Ntrk1*, PEP2 *Fam19a1*, and cold *Trpm8* (Figure 2E). Compared with recent snRNA-seq human data (Nguyen et al., 2021; Tavares-Ferreira et al., 2022), interesting differences in subtype identification were observed. For example, the H10 cluster in the study of Nguyen et al. (2021) and the pruritogen receptor cluster in the study of Tavares-Ferreira et al. (2022) mapped to both NP1 and NP2 in their study. Additionally, they observed the expression of known C-LTMR-specific markers including *TAFA4* and *P2RY1* in human C-LTMR, which were absent in putative C-LTMR in the study of Tavares-Ferreira et al. (2022). This may result from the most important limitation in the study of Tavares-Ferreira et al. (2022) that the resolution of spatial transcriptomic approach is lower than that of single neuron transcriptomes.

They also investigated correlations between the transcriptomes across species (Jung et al., 2023). The results showed that correlation between transcriptomes across all subtypes was highest between humans and cynomolgus monkeys. Humans and mice shared the most correlations between transcriptomes of proprioceptors and Aβ SA-LTMRs, but had the least correlations between the transcriptomes of the NP1 and PEP2 Fam19a1 subclusters. They further examined the expression profiles of classical genes, including ion channels, GPCRs, and neuropeptides. Divergent expression patterns for several genes were observed across species. For example, *TRPV1* was expressed more broadly in human DRG subtypes than that in mouse, guinea pig, and cynomolgus monkey. *SCN8A* was expressed almost in all DRG subtypes of primates whereas *SCN8A* selectively expressed in mouse LTMRs and PEPs. *CALCA* was more widely expressed in primates, whereas *CALCA* was more selectively enriched in mouse PEPs. Finally, they found that *TAFA4*, which is being evaluated for use in a clinical setting (Yoo et al., 2021), was expressed in distinct DRG subpopulations across species. For instance, *TAFA4* is predominantly expressed in C-LTMRs and some NPs in mice, whereas *TAFA4* is highly expressed in human Aδ-LTMRs, cold sensing neurons, and C-LTMRs. Therefore, *TAFA4* may serve as a molecular target for drug development with respect to specific sensory types.

## Similarities or differences between different studies in terms of the clustered found

Although increasing number of studies have attempted to classify DRG neurons at single-cell resolution, the heterogeneity in sequencing methods, laboratory protocols, or sample treatments between studies led to some divergent results. In general, DRG neurons across species were classified into PEPs and NPs (Usoskin et al., 2015; Hu et al., 2016; Renthal et al., 2020; Kupari et al., 2021; Wang et al., 2021; Zhang et al., 2022; Zhou et al., 2022; Jung et al., 2023; Yu et al., 2023), NFs (Usoskin et al., 2015; Renthal et al., 2020; Zhang et al., 2022), and C-LTMRs (Usoskin et al., 2015; Hu et al., 2016; Li et al., 2016; Renthal et al., 2020; Kupari et al., 2021; Tavares-Ferreira et al., 2022; Zhou et al., 2022; Jung et al., 2023; Yu et al., 2023) in most included studies of this review. Other common neuronal subtypes, such as TRPM8 (Kupari et al., 2021; Zhou et al., 2022; Jung et al., 2023; Yu et al., 2023), SST (Renthal et al., 2020; Zhou et al., 2022; Jung et al., 2023; Yu et al., 2023), proprioceptors (Usoskin et al., 2015; Hu et al., 2016; Tavares-Ferreira et al., 2022; Jung et al., 2023; Yu et al., 2023), Aβ-LTMR (Renthal et al., 2020; Tavares-Ferreira et al., 2022; Jung et al., 2023; Yu et al., 2023), Aδ-LTMR (Tavares-Ferreira et al., 2022; Jung et al., 2023; Yu et al., 2023) were commonly identified in several studies. Nevertheless, some subclusters were specifically reported in individual studies, such as MHNs and MNs (Li et al., 2016), TH (Usoskin et al., 2015), hATF3, hPIEZO, and hKIT (Yu et al., 2023). Interestingly, in the study of Yu et al. (2023), human SST^+^ populations belong to PEPs, whereas Jung et al. (2023) found that human SST clusters were NPs. Intriguingly, both NP1 and NP2 in the study of Jung et al. (2023) matched with the H10 cluster in Nguyen et al. (2021) and pruritogen enriched receptor cluster in Tavares-Ferreira et al. (2022). These inconsistent DRG subtype nomenclature across studies influence the comparing, integrating, and interpreting of gene expression profiles across species, and hinder the successful clinical translation. Future studies are required to directly determine the species differences under the equal conditions, such as same laboratory protocols and sample processing. Detailed information for each included study was summarized in Table 1.

**TABLE 1:** Study characteristics (including classifications from naive and nerve-injured DRGs).

References	Species	Sex	Age	Sample number	Location	Techniques	Gene numbers per neuron	Clusters number	Functional aspects
Usoskin et al., 2015	C57BL/6 mice	Male and female	6–8 weeks old	6	L4-L6	Single-cell RNA-seq	3,574 ± 2,010	11	3 LTMRs (NF1, NF2, NF3), 2 proprioceptive (NF4, NF5), and 6 types of thermosensitive (PEP1), itch sensitive (NP1, NP2, NP3), type C-LTMRs (TH) and nociceptive neurons (PEP2)
Li et al., 2016	C57BL/6 mice	Male	8–10 weeks old	5	L5	Single-cell RNA-seq	10,950 ± 1,218	10	6 small neurons types: 1 C-LTMR and 5 MHNs; 4 large neurons types: neurexophilin 1-expressing MHNs and MNs expressing Baiap2l1
Wang et al., 2021	C57BL/6 mice	Male	7–8 weeks old	6	L4-L5	Single-cell RNA-seq	6,407	19	6 PEP clusters, 5 NP clusters, 5 myelinated neurons types and 3 SNIICs
Renthal et al., 2020	C57BL/6 mice	Male and female	8–12 weeks old	5 male, 2 female	L3-L5	Single-nucleus RNA-seq	1,284	10	PEP1, PEP2, NPs, SST, Aβ-LTMRs, NF1, NF2, NF3, cLTMR, p_cLTMR2
Hu et al., 2016	C57BL/6 mice	Male and female	2 months old	NA	L3-L5	Single-cell RNA-seq	7,569 ± 1,456	5	NP, C-LTMR, PEP, LTMRs, proprioceptors
Zhang et al., 2022	C57BL/6 mice	Male and female	7–8 weeks old	5	L4-L5	Single-cell RNA-seq	NA	16	NF1-2, NP1-3, PEP1-6, cLTMR, CCI-ind1-4
Zhou et al., 2022	Sprague-Dawley rats	Male	Adult	24	L5	Single-cell RNA-seq	NA	11	NP, PEP, SST, C-LTMR, and TRPM8
Nguyen et al., 2021	Human donors	Male and female	34–55 years old	1 male, 4 female	L4-L5	Single-nucleus RNA seq	2,839 ± 1,917	15	NA
Tavares-Ferreira et al., 2022	Human donors	Male and female	24–65 years old	4 male, 4 female	Lumbar	Spatial transcriptomics	24,000 genes per section	12	5 C nociceptors, 1 C-LTMR, 1 Aβ nociceptor, 1 Aδ HTMR, 2 Aβ LTMRs, 1 Aδ LTMR and 1 proprioceptor subtypes
Yu et al., 2023	Human donors	Male and female	23–61 years old	1 male, 2 female	Thoracic (T11-T12) and L2-L5	Single-soma RNA-seq	9,486	16	hTRPM8, hC.LTMR, hNP1, hNP2, hPEP.SST, hPEP.TRPV1/A1.1, hPEP.TRPV1/A1.2, hPEP.PIEZO^h^, hPEP.KIT, hPEP.CHRNA7, hPEP.NTRK3, hPEP.0, hAδ.LTMR, hAβ.LTMR, hPropr, hATF3
Kupari et al., 2021	Rhesus macaques	Male and female	5–14 years old	1 male, 2 female	Lumbar	Single-cell RNA-seq	5,687	9	NP1, NP2, NP3, PEP1, PEP2, PEP3, C-LTMRs, TrpM8^high^, and A-LTMRs
Jung et al., 2023	C57BL/6J mice, guinea pigs, cynomolgus monkeys, and human donors	Male and female for humans and mice; only female for guinea pigs and monkeys	Mice: 6–17 weeks old; Humans: 21–48 years old; Guinea pigs: 5.5–6 months old; Monkeys: 8–9 years old	5 mice, 2 guinea pigs, 3 monkeys, and 7 humans (5 male and 2 female)	Lumbar	Single-nucleus RNA-seq	NA	11	Proprioceptor & Aβ SA-LTMR, Aβ SA-LTMR Ntrk3, Aδ-LTMR Ntrk2, C-LTMR P2ry1, NP1 Gfra1 Gfra2, NP2 Gfra1, NP3 Sst, PEP1 Adcyap1, PEP2 Ntrk1, PEP2 Fam19a1, cold Trpm8.

LTMRs, low-threshold mechanoreceptors; C-LTMRs, C-fiber low-threshold mechanoreceptors; MHNs, mechanoheat nociceptors; MNs, mechanical nociceptors; PEP, peptidergic; NP, non-peptidergic; SNIICs, spared nerve injury-induced neuron clusters; SST, somatostatin^+^ pruriceptors; NF, neurofilament; Aβ-LTMRs, Aβ low-threshold mechanoreceptors; cLTMR1, C-fiber LTMRs; p_cLTMR2, putative cLTMR2; Baiap2l1, BAI1-associated protein 2-like 1; TRPM8, transient receptor potential cation channel subfamily M member 8; NA, not available.

## New insights into the biological function of diverse somatosensory neurons in primate DRG

Bulk RNA-sequencing or proteomics analysis have revealed some expression changes of the whole DRG neurons and provide insights into the molecular mechanism for somatosensory disorders, such as chronic pain (Schwaid et al., 2018; North et al., 2019; Hall et al., 2022; Ray et al., 2023). However, bulk RNA-sequencing or proteomics analysis lack the functional information of specific neuronal subtypes, which can be resolved by single-cell/nucleus RNA-seq. Currently, the knowledge about biological function of diverse somatosensory neurons subtypes in primates is less known than that in rodents. Recent studies have identified the subclusters in primate DRG neurons (Kupari et al., 2021; Nguyen et al., 2021; Jung et al., 2023), and further predict and/or validate their functional profiles, which provides new insights into the biological function of diverse primate somatosensory neurons. As examples, *TRPM8* cluster (previously known as a receptor for cold temperature in mice) was suggested to function in both cold and heat temperature in human DRGs (Yu et al., 2023). The transcriptional profiles indicated hPEP.TRPV1/A1.1 senses cutaneous thermal and chemical nociceptive signals, and hPEP.TRPV1/A1.2 serves as viscera- and/or deep tissue-innervating chemical nociceptive afferents (Yu et al., 2023). Some hPEP.PIEZOh afferents were supposed to innervate blood vessels and perceive the blood pressure or flow, while some hPEP.PIEZO afferents might innervate visceral organs and sense non-noxious mechanical forces, such as regulating urination reflexes (Yu et al., 2023). The hPEP.KIT cluster probably serves as fast-conducting mechano-nociceptors (Yu et al., 2023). hPEP.CHRNA7 might be associated with deep pressure sensation (Yu et al., 2023). hC.LTMRs likely sense innocuous affective touch (Yu et al., 2023). hNP1 population likely responses to cowhage and mechanical forces (Yu et al., 2023). hNP2 and hPEP.SST clusters are likely histamine-sensitive but mechano-insensitive itch-sensing C-fibers (Yu et al., 2023). Kupari et al. (2021) identified the neuronal subtypes associated with human chronic pain. Their findings revealed that the heritability of multiple chronic pain conditions was significantly enriched in PEP1 clusters, including headaches, facial, neck and shoulder, stomach, and hip pain. While NP2 cluster was associated most significantly with the heritability of chronic back pain and hip pain. These new insights about the biological function of diverse somatosensory neurons in primate DRG will be important for development of novel therapeutic drugs for somatosensory disorders.

## Perspectives

The heterogeneity of neuronal composition has been well identified in both primates and rodent DRG by snRNA-seq and/or scRNA-seq. Meanwhile, the putative biological functions of many neuronal clusters have been predicted. However, the exact function of multiple clusters, especially in humans, have not been validated using *in vivo* experiments. Importantly, the critical genes for somatosensory disorders that are differentially expressed between human and rodents are urgently required to be identified, which will be of great importance for successful translation from rodents model to humans. It will also be interesting to identify the cellular heterogeneity and the specific function of DRG glial cells at single-cell resolution, as well as the interaction between neuron-glia, which also suggest to play an important role in somatosensory sensation or diseases (Tsuda, 2018; Hasel et al., 2021; van Weperen et al., 2021).

## Author contributions

All authors listed have made a substantial, direct, and intellectual contribution to the work, and approved it for publication.
